# Structural analysis of biological targets by host:guest crystal lattice engineering

**DOI:** 10.1038/s41598-019-51017-y

**Published:** 2019-10-23

**Authors:** Patrick Ernst, Andreas Plückthun, Peer R. E. Mittl

**Affiliations:** Department of Biochemistry, University of Zürich, Winterthurerstrasse 190, CH-8057 Zürich, Switzerland

**Keywords:** Biophysical chemistry, Proteins, X-ray crystallography, Protein design

## Abstract

To overcome the laborious identification of crystallisation conditions for protein X-ray crystallography, we developed a method where the examined protein is immobilised as a guest molecule in a universal host lattice. We applied crystal engineering to create a generic crystalline host lattice under reproducible, predefined conditions and analysed the structures of target guest molecules of different size, namely two 15-mer peptides and green fluorescent protein (sfGFP). A fusion protein with an N-terminal endo-α-N-acetylgalactosaminidase (EngBF) domain and a C-terminal designed ankyrin repeat protein (DARPin) domain establishes the crystal lattice. The target is recruited into the host lattice, always in the same crystal form, through binding to the DARPin. The target structures can be determined rapidly from difference Fourier maps, whose quality depends on the size of the target and the orientation of the DARPin.

## Introduction

Three-dimensional structural information is key for the understanding of almost any molecular process in life sciences. Furthermore, it is now an integral part of drug design. Diffraction methods are the prevailing techniques to obtain such information, but the scattering of photons by single molecules is too weak for direct recording. Scattering molecules need to be packed into well-ordered three-dimensional arrays, i.e. macromolecular crystals, to amplify the diffracted waves. Particularly for biological macromolecules, the conditions to favour crystallisation over aggregation are unpredictable and to find them a labour- and time-consuming trial-and-error process is required.

Since the early days of genetic engineering, mutagenesis methods have been used to improve the likelihood of achieving the crystalline state^1^, and/or the homogeneity of the crystalline molecules (reviewed in refs^2,3^). However, screening of constructs and crystallisation conditions is still required and successful crystallisation is not guaranteed. The unpredictable quest for suitable crystallisation constructs and conditions still limits protein crystallography, making fast, routine and predictable crystallisation systems highly desirable, ideally in combination with fast phasing by difference Fourier methods. While great progress in electron microscopy^4^ paves the way to atomic resolution structures without the crystallisation bottleneck, it is still a rather laborious undertaking. The structure determination by X-ray crystallography using a host:guest approach, perhaps similar to the workflow presented below, could be much faster and more suitable for high-throughput approaches.

**Figure 1:**
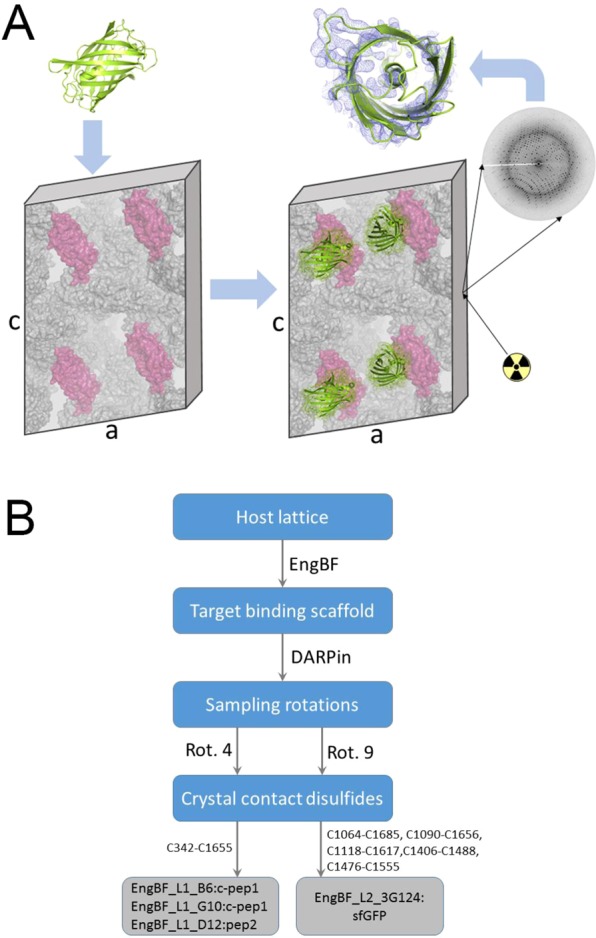
The concept of host lattice display and the design strategy. (**A**) The host lattice (surface representation) orients the target molecule (green cartoon) and permits amplification of diffraction. (**B**) Flow-diagram of the design strategy. Final constructs are shown as grey boxes.

Placing the target molecule at well-defined positions in an existing host lattice would be one option to crystallise arbitrary biological macromolecules under predefined conditions (Fig. 1A). This idea is not new; it was pioneered in DNA crystallography (reviewed in ref.^5^), even though to create sufficient order for high resolution may still present a challenge for DNA. It was also tested for small molecule crystallography^6,7^: in the ‘metal sponge’ technique, target molecules with low molecular weights were soaked into crystalline frameworks of porous metal complexes and the structures were determined by X-ray diffraction. Porous crystals of a putative polyisoprenoid-binding protein from *Campylobacter jejuni* have been used as a host lattice to study the absorption and release of fluorescent proteins and gold nano-clusters^8,9^, but crystal structures were only determined for small compounds after covalent attachment to the host lattice^10^. In summary, several strategies to use preformed crystals to overcome the crystallisation bottleneck exist, but none of them has been successful in determining the structures of larger guest molecules.

Here, we show how peptide and protein targets can be reproducibly crystallised as “guests” in a host crystal under predefined conditions, and we explain the advantages and current limitations of the host lattice-display method. In order to apply this approach to biological macromolecules, we engineered a host lattice comprising auxiliary- and target-binding domains; Fig. 1B outlines the design strategy.

## Results

The auxiliary domain defines the robust host lattice by providing the majority of crystal contacts, and thus also the crystallisation conditions. It serves as a scaffold for rigidly positioning the target-binding domain to immobilise guest molecules at well-defined positions. A suitable auxiliary protein needs to fulfil several premises: (i) It must form a stable crystal lattice with large solvent channels that resists perturbation by target protein binding. (ii) It must diffract X-rays to high resolution with and without guest molecule, to allow an accurate and rapid structure determination by difference Fourier analysis. (iii) It must be easily manipulated, expressed, purified and crystallised under mild conditions to maintain the integrity of the host:guest complex.

Although high solvent content and strong diffraction are usually orthogonal features of protein crystals (reviewed in ref.^11^), we identified several natural proteins in the PDB database that fulfil the requirements and could serve as auxiliary domains (Table 1). The most promising candidate, endo-α-N-acetylgalactosaminidase from *Bifidobacterium longum* JCM1217 (EngBF, PDB ID: 2ZXQ), is a 150 kDa protein (devoid of domain 1, UniProtKB Q3T552, residues 340 to 1694) that diffracts to 2 Å resolution and crystallises with 72% solvent in space group P6_5_ at neutral pH (25% 2-methyl-2,4-pentanediol (MPD), 3% PEG 20,000, 0.2 M NaCl, 0.01 M MnCl_2_, 0.1 M MES at pH 6.9)^12,13^. An additional carbohydrate-binding module (CBM32, not resolved in the electron density (ED) map) is connected to EngBF via a helical bundle whose C-terminus faces the large solvent-filled channel^13^. We replaced the CBM32 domain, after residue 1520 of EngBF, with different target-binding domains through rigid shared-helix fusions, similar to the design of various crystallisation chaperones^14,15^ and electron microscopy aids^16,17^.

**Table 1:** Suitable auxiliary domains for crystal lattice engineering.

V_M_ (Å^3^/Da)	Protein name	Expression system	Resolution (Å)	PDB
5.50	Mannosylglycerate synthase	*E*. *coli*	1.95	2BO4
4.77	Dipeptide epimerase	*E*. *coli*	1.90	3DER
4.74	Endo-1,4-beta-D-xylanase	*E*. *coli*	1.90	2W5F
4.71	Astrovirus serine protease	*E*. *coli*	2.00	2W5E
4.53	Argininosuccinate synthetase	*E*. *coli*	1.95	1KOR
4.50	Beta-galactosidase	*Penicillium sp*.	1.90	1TG7
4.46	Arylesterase	*E*. *coli*	1.65	3IA2
4.38	Endo-alpha-N-acetylgalactosaminidase	*E*. *coli*	2.00	2ZXQ

**Figure 2:**
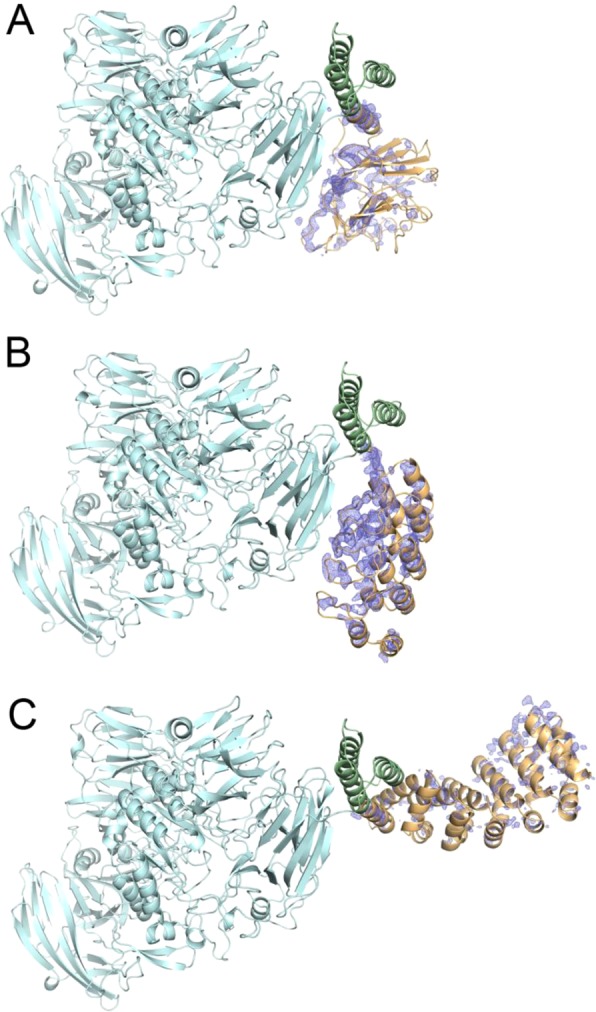
EngBF fused to various target-binding domains. EngBF fused to (**A**) B30.2 domain, (**B**) to DARPin domain, and (**C**) to dArmRP domain. EngBF domain, three-helix bundle and target-binding domain are shown in light blue, green and orange, respectively. All *2m*F_o_ − *D*F_c_ electron density maps were contoured at 1.0 σ.

First, we tested whether the EngBF lattice tolerates the insertion of target-binding domains. For this reason, we tested a designed Armadillo repeat protein (dArmRP, 329 residues)^18,19^, the B30.2 domain from sRFPL1 (201 residues)^20^, and a designed ankyrin repeat protein (DARPin, 162 residues)^21^. Suitable scaffolds must be small and rigid to fit in the solvent channel, expose a large paratope to lock the target molecule in a unique conformation, and their N-terminus should be α-helical to permit the rigid fusion concept using a shared helix^14,15^. All fusions crystallised isomorphically and crystals diffracted between 1.8 Å and 3.0 resolution, proving the feasibility of the fusion approach (Fig. 2). Yet, no continuous ED was visible for the fused domains, suggesting an inherent disorder of the target-binding domains in the host lattice, requiring further engineering.

**Figure 3:**
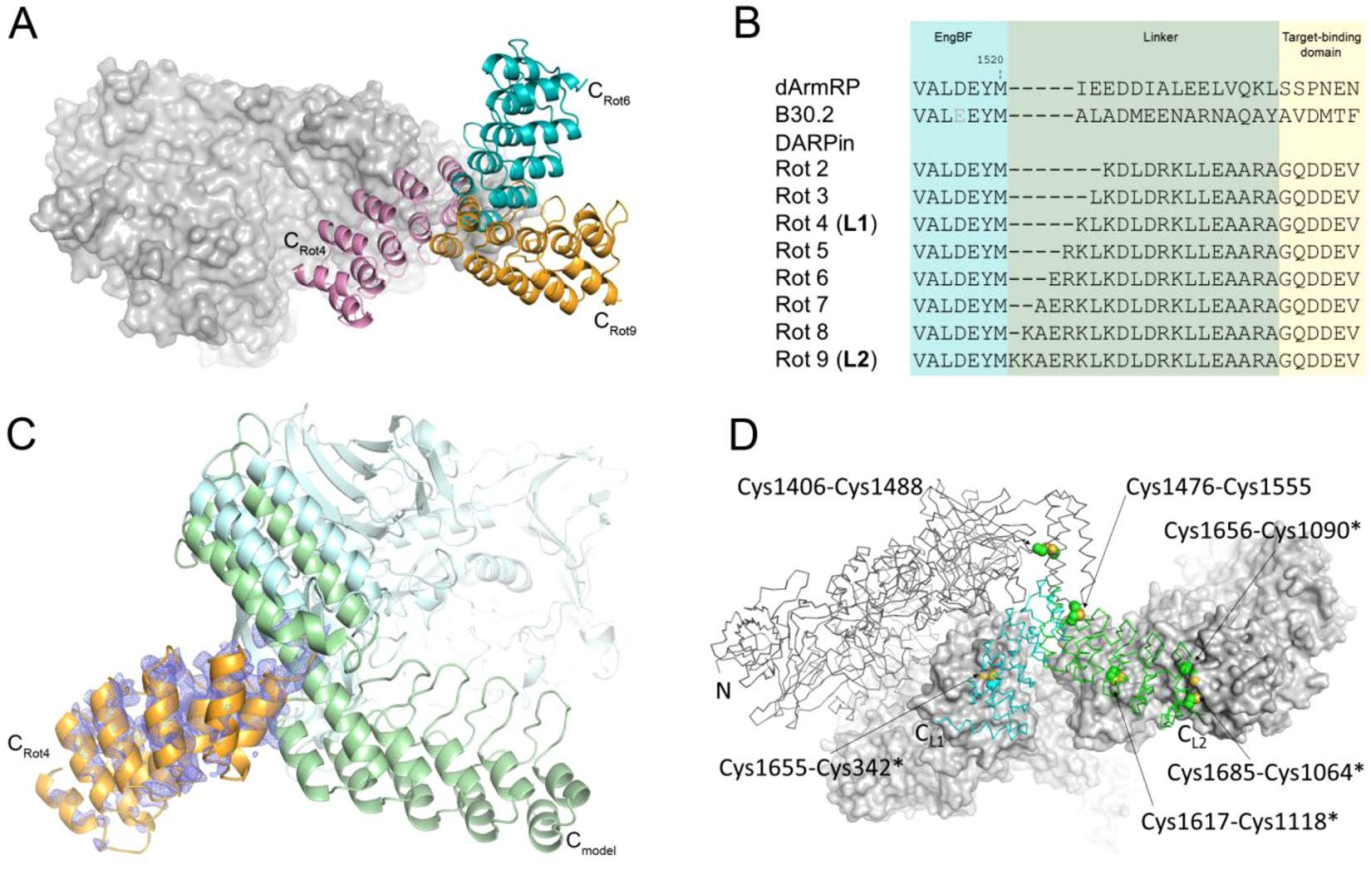
Design of constructs **L1** and **L2**. (**A**) Overlay of three DARPin-domain orientations viewed along the shared helix. The EngBF auxiliary domain is shown as a grey surface and DARPin rotations 4, 6 and 9 are highlighted as cartoons in pink, blue, and orange, respectively. Rotations were generated by stepwise extension of the helix linker between the EngBF and the DARPin-domain. (**B**) Alignment of linker helices that are shared between EngBF and target-binding domain. (**C**) Experimental structure of EngBF_DARPin_rot4 (light blue and orange cartoon) superimposed on the design model (dark green cartoon, the EngBF domain has been omitted for clarity). The *2m*F_o_ − *D*F_c_ map was contoured at 1 σ showing partial density for the C-terminal DARPin. In the experimental crystal structure, the three-helix bundle was shifted, causing a partial unwinding of the connecting helix and a 120° rotation of the DARPin domain. (**D**) Overview of inserted disulfide bridges. The EngBF-DARPin fusion is shown as a Cα-trace with the EngBF domain in grey and the DARPin domain in light blue (construct L1) and green (construct L2). Symmetry-related molecules are shown as molecular surfaces and disulfide bridges as spheres. Termini and disulfide bridges are labelled.

During the second design cycle, we rotated the DARPin domain in different orientations by stepwise extending the helical linker as shown in Fig. 3A,B. We proceeded with DARPins, as they are more rigid than dArmRPs, which possess some internal flexibility^22^. Furthermore, the selection of tight binders from DARPin libraries against almost any target molecule is well established^23^. Except for the structure EngBF_DARPin_rot4 (Table 2), where the shared helix was broken and a new crystal contacts formed via the DARPin paratope (Fig. 3C), the DARPin domains were still invisible in the ED maps. Again, this result confirmed that the DARPin fusions crystallised easily under the established conditions, but to have sufficient ED, additional crystal contacts were mandatory. These were engineered by incorporating disulphide bridges in the third design cycle.

**Table 2:** Data collection and refinement statistics for EngBF fusion structures.

Structure	EngBF_DARPin_rot4	EngBF_L1_B6:c-pep1	EngBF_L1_G10:c-pep1	EngBF_L1_D12:pep2	EngBF_L2_3G124oc
PDB-ID	4QEP	4QEV	6QFK	6SH9	6QFO
**Crystallisation**					
Precipitant	24.1% MPD, 4.2% PEG 20,000	26.3% MPD, 2.6% PEG 20,000	26.3% MPD, 2.6% PEG 20,000	25.9% MPD, 2.8% PEG 20,000	25.2% MPD, 3.4% PEG 20,000
Salt	200 mM NaCl, 10 mM MnCl_2_	200 mM NaCl, 10 mM MnCl_2_	200 mM NaCl, 10 mM MnCl_2_	200 mM NaCl, 10 mM MnCl_2_	200 mM NaCl, 10 mM MnCl_2_
Buffer	0.1 M MES NaOH pH 6.1	0.1 M MES NaOH pH 6	0.1 M MES NaOH pH 6.6	0.1 M MES NaOH pH 6.1	0.1 M MES NaOH pH 6.9
**Diffraction data**					
Resolution range (Å)	46.28–2.6 (2.693–2.6)	44.39–2.7 (2.797–2.7)	46.33–2.0 (2.072–2.0)	48–2.4 (2.486–2.4)	46.47–2.3 (2.382–2.3)
Space group	P6_5_	P6_5_	P6_5_	P6_5_	P6_5_
Unit cell (Å)	192.69, 192.69, 123.94	194.77, 194.77, 123.71	192.893 192.893 122.922	192.01 192.01 122.05	193.47, 193.47, 123.77
Total Reflections	1675848 (170899)	1284515 (130164)	3662645 (373197)	1041301 (102109)	2499741 (257012)
Unique reflections	80365 (8021)	73157 (7246)	174930 (17445)	99818 (9913)	116670 (11634)
Multiplicity	20.9 (21.3)	17.6 (18.0)	20.9 (21.4)	10.4 (10.3)	21.4 (22.1)
Completeness (%)	99.91 (99.93)	99.83 (99.83)	99.98 (99.99)	99.96 (99.96)	99.91 (99.75)
I/σ(I)	16.78 (1.14)	8.59 (0.80)	13.46 (1.22)	8.8 (0.93)	14.40 (0.68)
Mosaicity (°)	0.074	0.052	0.052	0.081	0.050
Wilson B-factor (Å^2^)	64.34	68.54	35.14	50.85	56.16
Rmerge	0.1678 (3.185)	0.3206 (2.944)	0.2476 (2.835)	0.2453 (2.553)	0.1867 (3.992)
Rmeas	0.172 (3.263)	0.3377 (3.03)	0.2538 (2.903)	0.258 (2.687)	0.1912 (4.085)
Rpim	0.03753 (0.7063)	0.08064 (0.7133)	0.0554 (0.6249)	0.07956 (0.8342)	0.04115 (0.8669)
CC1/2	0.999 (0.524)	0.994 (0.37)	0.998 (0.418)	0.995 (0.28)	0.999 (0.395)
**Refinement**					
Refl. for refinement	80329 (8021)	73127 (7243)	174871 (17445)	99801 (9912)	116569 (11609)
Refl. for Rfree	4017 (401)	3657 (362)	8744 (873)	4989 (496)	5830 (581)
R-work	0.177 (0.2969)	0.1778 (0.3118)	0.1526 (0.2819)	0.1633 (0.2967)	0.1749 (0.3363)
R-free	0.215 (0.3375)	0.2158 (0.3515)	0.1741 (0.3000)	0.1921 (0.3509)	0.2058 (0.3363)
RMS-bonds (Å)	0.010	0.010	0.010	0.012	0.009
RMS-angles (°)	1.23	1.26	1.10	1.75	1.12
Ramachandran plot (%)					
Favoured	94.92	95.06	96.24	96.13	96.14
Allowed	4.63	4.57	3.34	3.64	3.71
Outliers	0.45	0.37	0.15	0.22	0.15
Rotamer outliers (%)	4.48	4.98	1.52	2.62	3.28
Clashscore	3.61	3.36	1.30	1.81	1.53
Average B-factor (Å^2^)	89.26	71.92	46.46	60.97	73.26
Non-hydrogen atoms	10896	10968	12243	11359	11376
Protein	10299	10441	10525	10414	10331
Ligand	24	28	80	24	24
Water	573	499	1638	921	1013

Values in parentheses show the data for the highest resolution shell.

**Figure 4:**
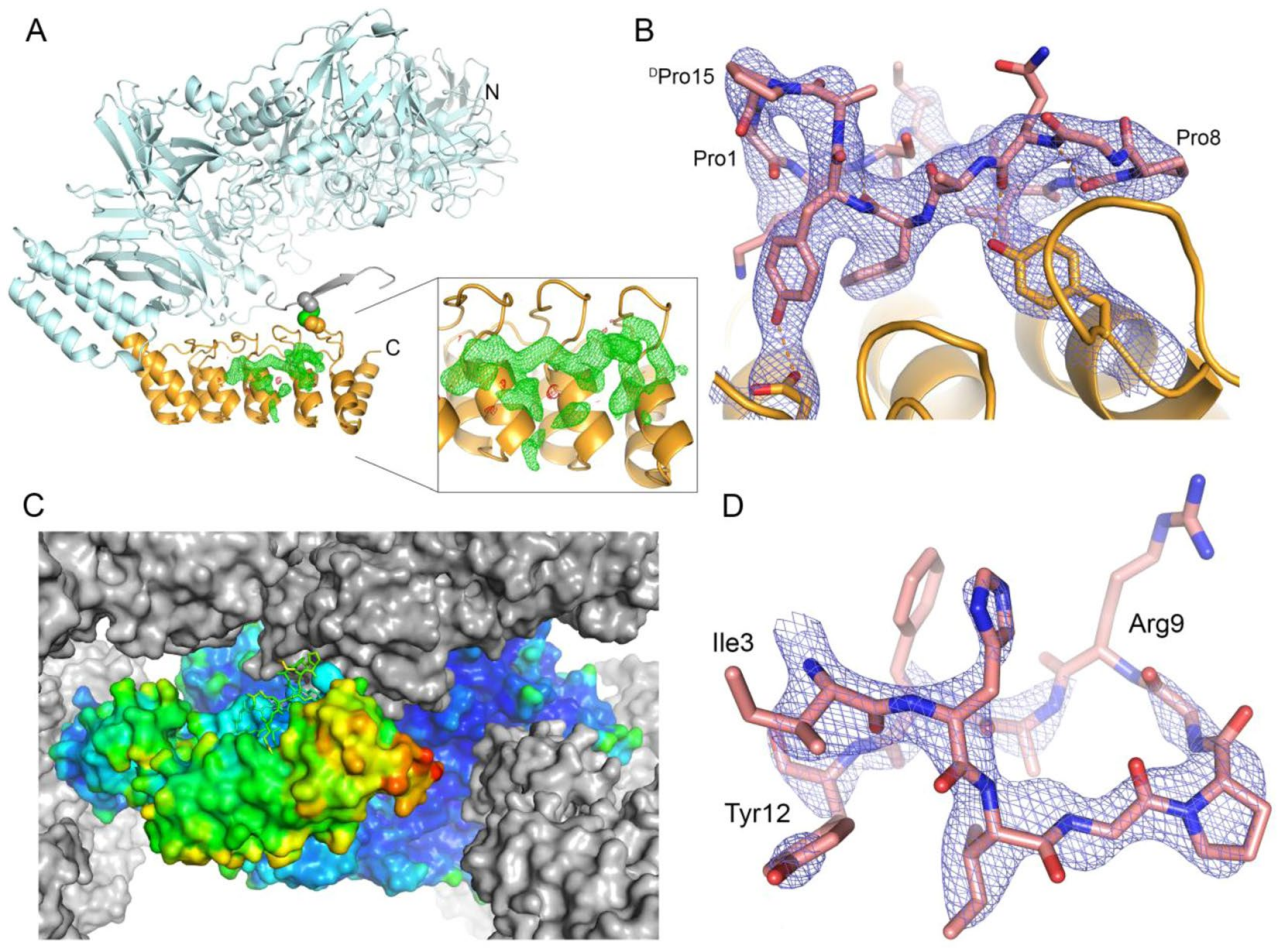
**L1** constructs with bound peptides. (**A**) Overview of EngBF-**L1**-DARPin_G10:c-pep1 complex. EngBF and DARPin_G10 are shown as a cartoon in light blue and orange, respectively, and the disulfide bridge Cys1655-Cys342* as spheres. The N-terminal beta-strand of the symmetry-related complex is shown in grey. The simulated annealing difference Fourier map is shown in green and red at contour levels of +3.5σ and −3.5σ, respectively. (**B**) Final *2m*F_o_ − *D*F_c_ map of peptide c-pep1 bound to EngBF-**L1**-DARPin_B6. The map was contoured at 1.1σ. (**C**) B-factor colouring of peptide c-pep1 (sticks) bound to EngBF-**L1**-DARPin_G10 (surface) with values ranging from 20 Å^2^ (blue) to 203 Å^2^ (red). Symmetry mates are shown as grey surfaces. (**D**) Structure of pep2 in complex with EngBF-**L1**-DARPin_D12. The *2m*F_o_ − *D*F_c_ map was contoured at 1.0 σ.

Based on the previous findings we selected two different orientations, **L1** from rotation 4 and **L2** from rotation 9, and introduced additional crystal contacts by inter-molecular disulfide bridges (Fig. 3D). EngBF-DARPin construct **L1** has a shorter shared helix, but the molecular packing only permits binding of small targets. To test if a single disulfide bridge stabilises the DARPin and allows target binding, we introduced mutations Lys1655 → Cys and Ser342* → Cys (*refers to a symmetry-related molecule) between the DARPin C-cap and the N-terminus of a symmetry-related EngBF domain. Using these mutations, DARPins B6 and G10 were fused to EngBF using the **L1** construct. These DARPins bind a cyclic peptide of 15 amino acids cyclised by a D-Pro-L-Pro unit (c-pep1)^24^. Complexes EngBF-**L1**-DARPin_B6:c-pep1 and EngBF-**L1**-DARPin_G10:c-pep1 co-crystallised under identical conditions as the native EngBF (Table 2) and diffracted to 2.7 Å and 2.0 Å resolution, respectively. The Cys1655-Cys342* disulfide bridge confers sufficient rigidity to identify the DARPin domain. The EngBF-**L1**-DARPin_B6 difference map was sufficiently clear to build residues 6 to 14 from peptide c-pep1 independently of prior structural knowledge (Fig. 4A), and the structure turned out to be virtually identical to the previously determined structure of DARPin_B6:c-pep1. After refinement, the c-pep1 main chain and most side chains were defined in the final ED map (Fig. 4B). The narrow space in the **L1** construct causes an additional crystal contact between c-pep1 and the host lattice (Fig. 4C and Table 3). This minor contact does not prevent crystallisation and may add additional stability to the design.

**Table 3:** Crystal contact and interface areas in host:guest complexes.

PDB	Complex	Guest		Host			
		Ident	Sym1	Sym1	Sym2	Sym3	Sym4
4QEV	Host: EngBF_L1_B6	716.5	3.2	1106.4	476.5	—	—
	Guest: c-pep1	—	—	(3.2)	—	—	—
4QFK	Host: EngBF_L1_G10	722.7	96.4	1173.5	508.0	—	—
	Guest: c-pep1	—	—	(96.4)	—	—	—
6SH9	Host: EngBF_L1_D12	479.9	—	1211.3	517.3	—	—
	Guest: pep2	—	—	—	—	—	—
6QFO	Host: EngBF_L2_3G124	—	—	829.2	478.5	541.3	27.4
	Guest: none	—	—	—	—	—	—

Surface areas in Å^2^ for polypeptide chains. Values in parenthesis are listed twice for completeness. Hyphens indicate the absence of contacts. Definition of symmetry operators: Ident: x, y, z; Sym1: −y, x − y − 1, z-1/3; Sym2: x − y, x, z − 1/6; Sym3: −x + 1, −y, z − 1/2; Sym4: x − y, x, z + 5/6.

To show that this strategy works for other small ligands as well, we inserted DARPin_D12 that recognises pep2, which also comprises 15 amino acids like c-pep1 but lacks the D-Pro-L-Pro unit. Again, crystals were obtained under the established conditions and the ligand was visible in the 2.4 Å resolution difference map. Here, residues 1, 2, 6, and 13–15 from pep2 are not resolved in the final ED map, suggesting that internal molecular rigidity, conferred by the cyclisation unit in c-pep1, is required to resolve the target completely (Fig. 4D). In summary, while the **L1** construct is useful for rapidly determining structures of small targets under predefined crystallisation conditions, it provides little space for larger targets.

In contrast to **L1**, the **L2** linker orients the DARPin paratope towards the central solvent channel of the EngBF lattice, allowing for larger targets to bind, but with fewer possible crystal contacts. For creating a very rigid **L2** fusion, a disulfide bridge between Val1406 → Cys and Thr1488 → Cys was used to shift the three-helix bundle and the connected DARPin closer to a symmetry-related EngBF domain. A second intramolecular disulfide bridge (Glu1476 → Cys to Glu1555 → Cys) connects the loop of the EngBF three-helix bundle with the loop between the DARPin N-cap and its first internal repeat to reduce bending motions (Fig. 3D). Three additional disulfides between the DARPin domain and a symmetry-related EngBF domain crosslink the DARPin in the crystal (Cys1064*-Cys1685, Cys1090*-Cys1656, Cys1118*-Cys1617). As a test, we inserted DARPin_3G124, a high-affinity binder for sfGFP^25^.

**Figure 5:**
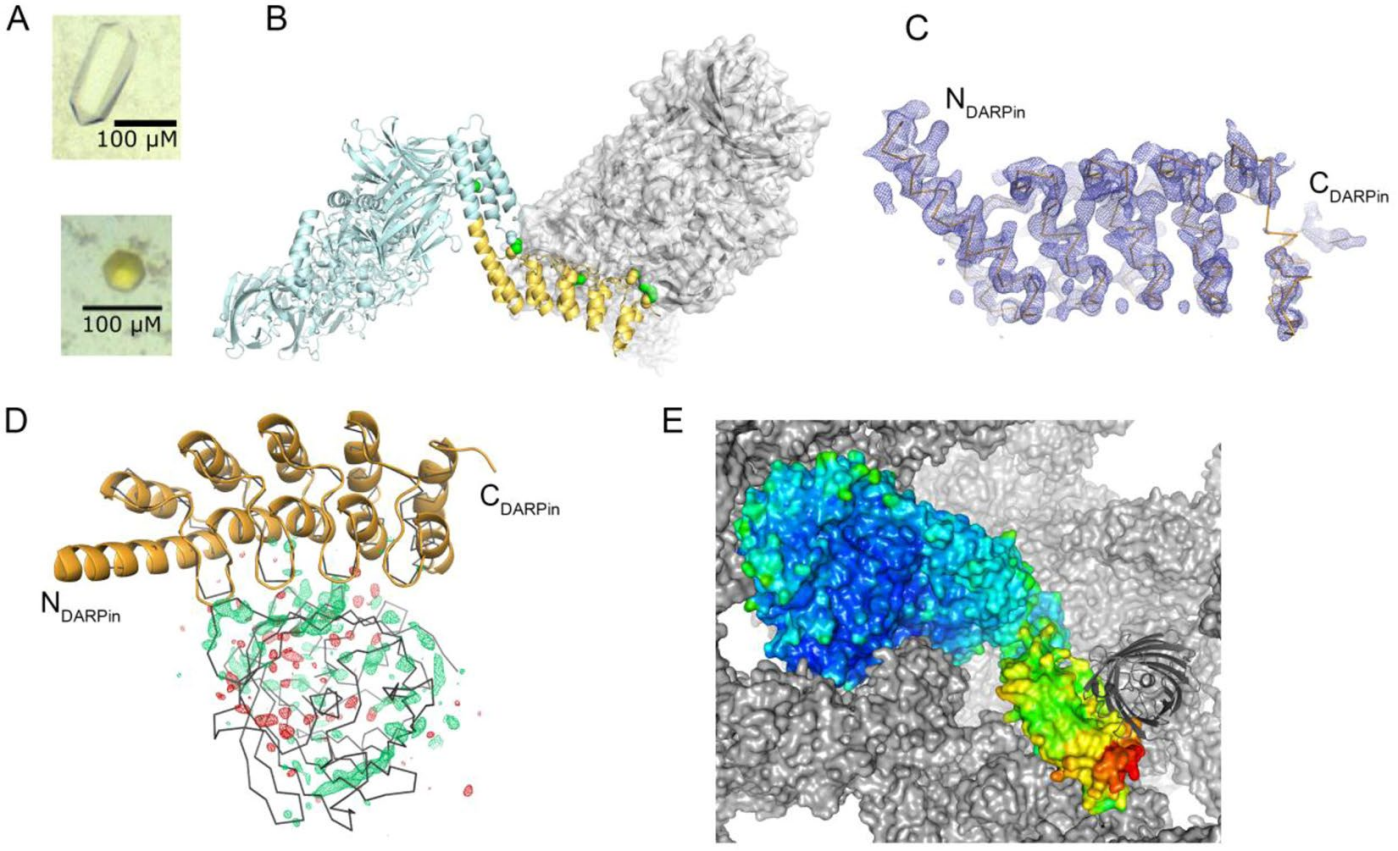
**L2** construct with DARPin_3G124. (**A**) Crystals of the EngBF-**L1**-DARPin_B6:c-pep1 complex (top) and EngBF-**L2**-DARPin_3G124 co-crystallised with sfGFP (bottom). (**B**) Structure of EngBF-**L2**-DARPin_3G124 (light blue for EngBF part and yellow cartoon for fused DARPin). Inserted cysteines are depicted as spheres and the symmetry-related molecule is in grey. (**C**) Final *2m*F_o_ − *D*F_c_ map of the DARPin_3G124 domain. The map was contoured at 1.0σ. (**D**) DARPin_3G124nc:sfGFP complex (PDB-ID 5MA6, grey Cα-trace) superimposed on EngBF-**L2**-DARPin_3G124 (orange cartoon). The *m*F_o_ − *D*F_c_ map was contoured at +3.1σ (green) and −3.1σ (red) and is shown around the sfGFP with an 8 Å cushion. (**E**) B-factor colouring of EngBF-**L2**-DARPin_3G124 (surface) with values ranging from 31 Å^2^ (blue) to 252 Å^2^ (red). Symmetry mates are shown as grey surfaces. The superimposed sfGFP is shown as a grey cartoon to indicate the position of the target.

EngBF-**L2**-DARPin_3G124 was co-crystallised with sfGFP, again under the established conditions and the yellow crystal colour suggested that sfGFP was absorbed in the EngBF-**L2**-DARPin_3G124 lattice (Fig. 5A). The EngBF-**L2**-DARPin_3G124 crystals diffracted to 2.3 Å resolution in the presence of sfGFP (Table 2) and the ED map confirms that the DARPin_3G124 domain is locked in the desired orientation with the paratope pointing towards the solvent-filled channel of the EngBF host lattice (Fig. 5B,C). This orientation provides sufficient space for larger targets up to 40 kDa, such as sfGFP. After refinement of EngBF-**L2**-DARPin_3G124 in the absence of the target, residual ED suggests binding of sfGFP, but the ED map is insufficient for placing sfGFP without additional information. Superposition of the DARPin_3G124nc:sfGFP structure (PDB-ID 5MA6^26^) on EngBF-**L2**-DARPin_3G124 reveals that the difference map agrees very well with the expected orientation of sfGFP (Fig. 5D). After placing the sfGFP based on the superimposed complex, the EngBF-**L2**-DARPin_3G124:sfGFP complex was refined at 2.3 Å resolution. Refinement of EngBF-**L2**-DARPin_3G124 free and in complex with sfGFP yielded very similar R_work_/R_free_ values of 0.175/0.206 and 0.171/0.205, respectively. After refinement, sfGFP possesses an elevated B-factor of 214 Å^2^ and the 2*m*F_obs_ – *D*F_model_ σ_A_-weighted map shows discontinuous density for the sfGFP main chain and no clear side chain density (data not shown). The B-factors vary along the main chain of all EngBF fusion proteins (Figs 4C and 5E). In all fusions EngBF, the robust scaffold of the crystal lattice, shows equally low B-factors, both by itself and in all refined fusion constructs, while the B-factors for the DARPin domains and for the targets are higher (Table 4). Since the B-factor for sfGFP exceeds 200 Å^2^ and due to the marginal contribution on the improvement of R_free_, we deleted the sfGFP chain from the final model.

**Table 4:** B-factor distribution in EngBF-DARPin fusions.

Complex	Temperature factor [Å^2^]		
	EngBF	DARPin	target
EngBF_DARpin_rot4	75.0	207.3	—
EngBF-L1-DARPin_B6:c-pep1	66.7	107.0	127.6
EngBF-L1-DARPin_G10:c-pep1	38.9	86.7	92.2
EngBF-L1-DARPin_D12:pep2	54.9	103.4	136.5
EngBF-L2-DARPin_3G124	63.1	150.6	—

Low RMSDs suggest that the crystal engineering approach has not perturbed the structures of the individual domains. We measured 0.20 Å and 0.72 Å for the superposition of the refined EngBF-**L2**-DARPin_3G124 structure on isolated EngBF (PDB-ID 2ZXQ, 7646 atoms) and DARPin_3G124nc (PDB-ID 5MA6, 869 atoms), respectively.

## Discussion

**Figure 6:**
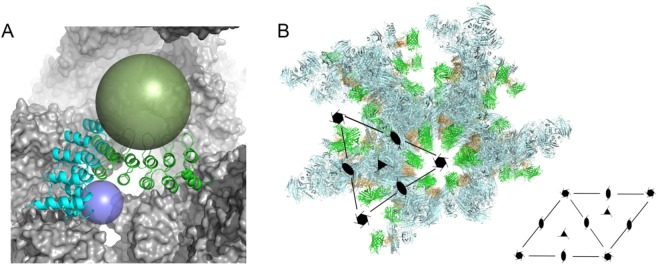
Host:target complexes. (**A**) Available space comparison for targets in constructs **L1** and **L2**, which are sketched as transparent spheres in blue (construct **L1**, 10 Å radius) and dark green (construct **L2**, 20 Å radius). The host lattice is shown as a grey surface and the DARPin domains for constructs **L1** and **L2** as cartoons in cyan and green, respectively. The view is similar to Fig. 3D. (**B**) Perspective view of the crystal arrangement with EngBF-**L2**-DARPin_3G124 in the crystal oriented along the P6_5_ symmetry axis. The symmetry elements are schematically drawn into the picture with the organisation of the unit-cell shown below the picture. EngBF-domain in pale-cyan, DARPin in orange, and the superimposed sfGFP (PDB-ID 5MA6) in green.

Our analysis shows that EngBF crystals – and perhaps other host crystals as well – tolerate the insertion of target:binder complexes and still robustly form under the same established crystallisation conditions. The target must be locked in a unique orientation to give a clear ED, which can be achieved by rigid and rigidly connected scaffolds such as e.g. the DARPins with a very constant geometry^23,27^. From one target to the next, only the binding residues of the DARPin need to be exchanged, as the shape of this binding molecule is very constant, and suitable DARPins can now be routinely selected for up to 95 targets in parallel. We created two different positions for guest molecules. In both cases target molecules line up along the central solvent channel, albeit with different orientations (Fig. 6A,B). Construct **L1**, with the DARPin domain facing a smaller cavity, can bind spherical targets with diameters up to 20 Å (targets below 3–4 kDa), whereas construct **L2** can recognise targets with diameters up to 40 Å (targets below 40 kDa) (Fig. 6A).

The confinement in **L1** offers sufficient rigidity to unambiguously refine the conformation of smaller targets, provided that the target itself possesses a rigid three-dimensional structure, whereas the extended space in **L2** comes at the expense of reduced molecular rigidity. The yellow colour of the EngBF-**L2**-DARPin_3G124 crystals in the presence of sfGFP confirms that the target penetrates the host lattice and adsorbs to a higher concentration than present in the mother liquor, but to judge how much they contribute to diffraction, pure adsorption is an insufficient criterion, because the target molecules must be oriented in a rigid conformation. The residual ED suggests that this is at least partially the case for sfGFP, but the refinement parameters, such as average B-factor and improvement in R_free_, indicate only a marginal contribution of sfGFP, which is currently insufficient for building an independent structural model.

The poor local resolution for sfGFP could either be due to low occupancy or thermal motions. In solution DARPin_3G124 binds sfGFP with a K_D_ of 22 ± 0.3 nM^25^. Since sfGFP was present during crystallisation at a concentration of 0.5 mg/ml (equivalent to approximately 16 μM, 5-fold molar excess over the EngBF-DARPin fusion, and thus 1000-fold above the expected K_D_) we can assume that the occupancy is high. As the sfGFP-DARPin complex structure shows the same interface in the EngBF complex as without EngBF, and since there are no clashes, it is reasonable to assume that the dissociation constants of the EngBF-**L2**-DARPin_3G124:sfGFP complexes in the crystal and in solution are intrinsically similar. Nonetheless, the crystallisation buffer and precipitant may influence the K_D_, and the crystal lattice could have distorted the interface, such that the occupancy might actually be lower than expected from the affinity and the concentrations used.

On the other hand, we observed a pronounced B-factor gradient ranging from below 40 Å^2^ for EngBF to above 150 Å^2^ for the DARPin domain and even higher for the target (Table 4). Typically, the B-factor of the DARPin domain is approximately twice as high as the B-factor for the EngBF domain and the B-factor of the target is always higher than the B-factor of the DARPin domain, because the DARPin domain provides the main lattice contacts for the target. For EngBF-**L2**-DARPin_3G124 we observed an average B-factor of 150.6 Å^2^ for the DARPin_3G124 domain. Due to the lack of additional crystal contacts, thermal motions of the target are restricted by the DARPin paratope only. The B-factor of the DARPin and the fraction of molecular target surface, which is buried in the DARPin interface, dictate the B-factor of the target and consequently the precision of its ED. Therefore, a B-factor exceeding 200 Å^2^ can be expected for sfGFP even at full occupancy in the **L2** construct. In the crystal of the individual DARPin_3G124:sfGFP complex (PDB-ID 5MA6) the average B-factors are 79.8 and 87.3 Å^2^, respectively, showing no intrinsic flexibility in this complex. We conclude that the rigid embedding of the target-binding domain, which is achieved by the shared helix and the engineered disulfide bridges in our case, is absolutely essential for host-lattice display to reveal sufficient ED for the target. In the future the engineering of additional crystal contacts of the target-binding domain and an extended paratope will be necessary to constrain the molecular order more effectively and to improve the ED for larger targets. This will be the prerequisite to make this approach truly generic.

A host:guest approach like this offers additional advantages. The intrinsic phase problem of X-ray crystallography is reduced to difference Fourier maps, making this technique particularly attractive for structures where simple phasing techniques like molecular replacement cannot be applied. Proteins with intrinsically disordered regions are notoriously difficult to crystallise, but since disorder does not hamper the selection of binders, the system presented here should allow the structural analysis of at least the rigid regions of the target molecule. The solvent channels of the host lattice permit easy access to the target and reduce the impact of crystal lattice forces on the conformation of the target, making this approach also attractive for drug design and time-resolved studies^28^.

In conclusion, this work has laid the foundation for a host:guest approach to protein crystallography, obviating the need to empirically search for crystallisation conditions. While this concept has been discussed for many years, this may be the first practical implementation for larger targets that can be extended into a general approach. Future designs will have to address the challenges of creating more anchor points to define the position and orientation of larger guests even better to improve the ED further.

## Materials and Methods

### Shared helix and disulfide design

Suitable host lattices were identified using the advanced search tool from the RCSB Protein databank internet service. The databank was queried for lattices with high solvent content and resolution. The results were manually curated in light of the molecular structure, to assess if the host lattice permits the fusion of target-binding domains.

Shared helices were designed according to ref.^15^ using the Rosetta molecular modelling suite^29^ for the dArmRP and the B30.2 fusions. For the DARPin fusions, shared helix H15flex from ref.^15^ was used as a template for the connection between EngBF and the DARPin. Potential disulfides where identified using the *Disulfide by Design Server 2*.0^30^.

### Cloning, expression and purification

DNA encoding different EngBF fusion constructs was cloned into a pQIq vector (a lacI^q^ encoding derivative of pQE30 (Qiagen, Hilden, Germany)), containing an N-terminal sfGFP fusion and a C-terminal His_6_-tag, both cleavable via a 3C protease cleavage site as described in ref.^26^. DNA fragments encoding the respective DARPin fusions with different binding sites and cysteine residues were ordered from IDT (Coralville, USA) or Genewiz (South Plainfield, USA) and cloned into the target vector via a BglII and a HindIII site. Chemocompetent *E*. *coli* BL21-Gold cells were transformed with the respective plasmid and used both for cloning and expression. Genes were expressed in 200–400 mL auto-induction 5052 medium^31^ for 15 h at 25 °C. The cells were subsequently harvested by centrifugation at 5,000 × g for 10–15 min and resuspended in 15–20 mL washing buffer (20 mM sodium phosphate pH 6.3, 200 mM NaCl, 20 mM imidazole) and lysed by sonication. Cell debris was centrifuged at 20,000 × g for 15–20 min and the supernatant was loaded on 5 mL NiNTA-agarose resin (Qiagen, Hilden, Germany). Columns were washed with 5 column volumes (cv) of washing buffer and protein was eluted using 10 mL elution buffer (20 mM sodium phosphate pH 6.3, 200 mM NaCl, 250–500 mM imidazole). The elution fraction was directly loaded onto 2 mL Sepharose resin coupled with DARPin clamp R7, which binds to GFP with picomolar affinity as described in ref.^26^. The resin was washed with 20 mL crystallisation buffer (20 mM sodium phosphate, pH 6.3, 200 mM NaCl). To cleave the EngBF fusion construct off the column, 2 mL crystallisation buffer containing 1 mg HRV 3C protease were loaded on the column. Cleavage was either carried out overnight at 4 °C or for three hours at 25 °C for constructs **L1** and **L2** containing the cysteine mutations. Cleaved protein and protease were subsequently washed off the GFP-binding column with 10 mL crystallisation buffer and washed through 2 mL Ni-NTA resin columns to remove the His_6_-tag peptide and the protease (also carrying a His-tag). Proteins were directly used for crystallisation and always freshly prepared.

### Crystallisation and structure determination

Proteins were concentrated to 2–20 mg/mL using Amicon^®^ centrifugal concentrators (50,000 MWCO, Merck Millipore, Massachusetts, USA)) and set up for crystallisation in a fine screen of the initial conditions (25% 2-methyl-2,4-pentanediol (MPD), 3% PEG 20,000, 0.2 M NaCl chloride, 0.01 M MnCl_2_, 0.1 M MES pH 6.9), changing the pH along the columns (from pH 6 to 7) and the MPD/PEG 20,000 ratio along the rows (MPD from 23% to 27% (v/v) and PEG 20,000 from 5% to 2% (w/v)) in a 96-well format. Three different mother-liquor to protein ratios (1:1, 2:1, 5:1) in 300–400 nL drops were used per well and incubated against 75 µL of reservoir solution at 4 °C. For **L1**/**L2** complex crystallisations, the ligand was added in two-fold (c-pep1) to five-fold (sfGFP) molar excess and incubated 1–3 h prior to setting up the crystallisation experiment.

Crystals grew between day 0 and day 25 and were flash-frozen in liquid nitrogen prior to data collection without any additional cryo-protectant. Diffraction data collection was done at 1 Å at beamlines X06SA or X06DA (Swiss Light Source, PSI, Villigen, Switzerland) equipped with an Eiger 16M or Pilatus 2 M detector (Dectris, Baden-Wättwil, Switzerland). Data collection and refinement statistics are summarized in Table 2. Data processing was done using XDS, XSCALE and XDSCONV^32^. To match the polar 6_5_-screw axis with the deposited diffraction data of EngBF (PDB-ID 2ZXQ) data were re-indexed using the operator (hkl) = (kh-l) if necessary. Structures were determined by difference Fourier analysis. Model building was done in Coot^33^ and refinement using REFMAC5^34^, PHENIX refine^35^ and BUSTER^36^. Final resolution of the datasets were determined by paired refinement in pdb_redo^37^ according to ref.^38^.

## Data Availability

All data needed to evaluate the conclusions in the paper are present in the main text or the supplementary materials. Plasmids encoding the constructs reported in this study are available for research purposes from the authors. Coordinates and structure factors have been deposited in the Protein Data Bank with the accession codes 6QFO, 6QFK, 6QEV, 6QEP, and 6SH9. Raw diffraction data are available at https://proteindiffraction.org/.
